# Attitudes toward Menopause in HIV-Infected Cambodian Women

**DOI:** 10.3390/healthcare9060677

**Published:** 2021-06-04

**Authors:** Roshna Thapa, Youngran Yang

**Affiliations:** 1 College of Nursing, Jeonbuk National University, Jeonju 54896, Korea; thaparashu14@gmail.com; 2 Research Institute of Nursing Science, Institute for Southeast Asian Studies, College of Nursing, Jeonbuk National University, Jeonju 54896, Korea

**Keywords:** Cambodia, HIV/AIDS, menopause, attitude

## Abstract

(1) Background: Attitudes toward menopause are believed to play a potential role in the experience of menopause, including its perceived severity. However, the studies available on the perspectives of women living with human immunodeficiency virus (HIV) on menopause in Cambodia are very limited. This study aimed to evaluate the attitudes toward menopause of Cambodian women living with HIV. (2) Methods: A cross-sectional study was conducted among 189 women using a questionnaire titled Attitude toward Menopause Scale and socio-demographics. (3) Results: The study analysis showed that the participants had slightly negative attitudes toward menopause with the mean attitude score of 86.81 ± 10.79 (Range 35–140). Postmenopausal women displayed more positive attitudes than premenopausal women. Older age, higher education, and a non-drinking habit were independently associated with a positive attitude toward menopause. (4) Conclusions: The results suggest the need for a multidisciplinary team of health care experts that would address the special needs of this population marked by the menopausal transition.

## 1. Introduction

Benefiting from the advancement and widespread commencement of antiretroviral regimens, a 25-year-old diagnosed with HIV from 2000 through 2005 is estimated to survive a median of 39 additional years; they can now form a long-term perspective plan for their healthy aging and may expect to be treated equally with respect to access to health services and counseling [1]. At the end of 2013, there were 4.2 million people aged 50 and older living with HIV; 13% of this adult population were aged 50 or older [2], and this is expected to increase to 21% by 2020 [3]. In low- and middle-income countries alone, the sub-population of people aged 50 and older represents approximately 12% of all the adult people living with HIV [4]. Among them, in Cambodia, 35,000 females over the age of 15 are HIV-infected, out of whom around 21% are aged 50 years or older [5]. The longer survival of HIV-infected people means that increased numbers of women living with HIV (WLHIV) are expected to enter and live beyond menopause.

Enhancing knowledge about menopause is necessary because the physiologic changes associated with menopause alter the short- and long-term quality of life of women, and the condition is even worse for WLHIV [6]. Menopause occurs earlier among WLHIV and they are likely to experience menopausal symptoms of greater frequency and intensity than their HIV-negative counterparts [7]. Not only this, menopause and HIV have additional effects on each other, further increasing the risk of other physiological disorders like cardiovascular diseases, progression of cervical dysplasia, and osteoporosis [8]. Hence, the health care demand of this population represents a relatively new challenge. However, evidence shows that the menopause of WLHIV has been considered merely as a chronic health focus and is ignored in the health care systems [9], especially in the developing countries.

Although menopause is a physiological event, it is also considered psychosocial because it is always surrounded by psychosocial phenomena like attitudes, perceptions, and expectations, which also differ across cultures and societies [10,11]. Many Nigerian women perceive it as a welcome relief from menses while others consider it an unwelcomed event [12]. Of the respondents in an American study, 81% agreed menopause to be one of the biggest changes in a woman’s life [13]. Attitudes toward menopause are believed to play a potential role in the event’s experience, including its perceived severity [14]. Positive attitudes toward menopause are closely associated with less severity of symptoms and better quality of life of women [10,14,15]. Assessing women’s attitudes toward menopause is an initial step for predicting the knowledge gap and may also aid in health talks and counseling within a cultural context.

After the pioneer study by Neugarten and colleagues in 1963 [16], attitudinal aspects of the menopausal transition have been frequently studied. However, most studies have targeted healthy women and have neglected to include midlife women with major illnesses of HIV/AIDS. To our knowledge, few researches on WLHIV such as on the effects of menopause on wellbeing [7], menopause and physical and psychological health [17], menopause and sexual activity [18], and menopausal symptoms in WLHIV [8,19] have been conducted. For the healthy aging of WLHIV, understanding their attitude toward menopause should be prioritized; however, very limited study is available on the perspective of WLHIV on menopause [20]. In the context of Cambodia, no study has yet explored the viewpoint of WLHIV regarding menopause. Menopause should be seen and understood socio-culturally by the values and worth placed by a particular society [21]. In this study, we explore, for the first time in Cambodia, what older Cambodian women with HIV think about menopause. The results here would help health care personnel better address the issue of menopausal health in a salient way for this population.

## 2. Materials and Methods

### 2.1. Study Participants and Data Collection

We conveniently sampled Cambodian women living with HIV ages 40 to 60 years from the HIV outpatient department of a hospital in Phnom Penh, the capital of Cambodia. Inclusion criteria included being able to answer the questionnaire, at least with the assistance of an interviewer, and seropositive status confirmed by enzyme-linked immunosorbent assay (ELISA) or Western blot. The exclusion criteria were: (1) pregnant, (2) breastfeeding mother, and (3) medically induced menopause. Institutional review board (IRB) approval from Human Subjects Committee of Jeonbuk National University, the Cambodia National Ethics Committee for Health Research, and the Ministry of Health, Cambodia, was obtained.

Participants underwent in-person interview in the local Khmer language on a one-on-one basis by a team of researchers including a doctor, nurse, and social worker of the same hospital. The team members were provided training on the purpose of the study, the contents and administration of the questionnaire, and how to approach the participants and document their responses accurately. All the caretakers from the hospital were gathered in a waiting room of the hospital and informed about the aim of the study; only those interested were enrolled for the study. Participants provided informed written consent prior to their interviews; participation of the respondents was on a voluntary basis. The interviewers read the questionnaire to the participants in the case of illiterate participants. Confidentiality was assured by closing all the doors and windows of the interview room. G*Power version 3.1.9.2 estimated the sample size at 167, considering a maximum of 19 sociodemographic variables, a level of significance of 0.05, statistical power of 0.95, and a small effect size of 0.2. However, to ensure accuracy, a further 22 samples were added. A total of 189 women completed the interview, with incomplete interview by 11 participants (5.5%). Participants were compensated with a small amount of money for the time spent on the interview.

### 2.2. General Characteristics

Baseline information was obtained from the respondents on demographic characteristics (e.g., age, residence, and education level), reproductive history (e.g., age of menarche and menopausal status), and disease/infection-related health characteristics (e.g., perceived health and life state, and conditions like hypertension). The HIV infection-related information about the patients such as duration of HIV, duration of antiretroviral therapy (ART), WHO HIV/AIDS clinical stage, and CD4 count were referred from the hospital records. Perceived life and health status were rated on a Likert scale of 0–10. Height and weight were self-reported by the participants which was cross-verified from the hospital records.

### 2.3. Menopausal Status

Premenopause was defined according to the Study of Women’s Health across the Nation (SWAN) guidelines [22] as menses in the previous three months without any irregularity. Early perimenopause was defined as menstrual bleeding in the previous three months but with increasing irregularity in cycle length over the past year, and late perimenopause was the absence of menses in the last three months but who had had menses in the previous 12 months. For this study, we did not distinguish between the two separate groups for perimenopause due to the small number of women in individual groups. Postmenopause was defined as no menses in the previous 12 months (not due to any medication, pregnancy, or severe weight loss).

### 2.4. Attitudes toward Menopause (ATM)

A modified version of the Neugarten ATM, 35-item (16 positive items and 19 negative items) scale was administered to assess the attitudes toward menopause [22]. For each item on the scale, participants were asked to indicate if they agree or not. All the responses were scored on a 4-point Likert scale ranging from 1 (disagree strongly) to 4 (agree strongly). Negative items were scored inversely and the higher scores indicated more positive attitudes. A total score, ranging from 35 to 140, was calculated by summing the scores of all the items. The mean was considered the final score and was calculated by dividing the total score by the number of items.

Since the tool was not in the local language, a Khmer version of this tool had to be developed. Using back-translation, two professional translators specializing in the health field created a Khmer translation from the original English version [23]. A panel of experts, including local women’s health doctors and nurses from a Phnom Penh AIDS hospital, then made some minor but necessary corrections, and confirmed the consistency of the two versions as well as the readability, language simplicity, and suitability of the Khmer version. A pilot study was performed on 30 women to assess its consistency and reliability, but they were not included in the main study. For the participants’ easy understanding, the phrase “the change of life” in the tool was simplified as “menopause”. The Cronbach’s alpha of the ATM scale for American women has been reported to be 0.64 [13], and 0.84 for Iranian women [24]; the Cronbach’s alpha for the current sample was 0.747.

### 2.5. Statistical Analysis

Frequency and percentages determined the general characteristics of the subjects. Bivariate analysis of the baseline characteristics was performed using ANOVA and Kruskal-Wallis with Duncan’s or *t*-test for each item. A *p*-value of 0.05 was considered statistically significant. Multiple linear regression analyses were performed to predict attitudes toward menopause and only those variables that were significant for the bivariate analysis were allowed to enter the equation as confounding variables. The data were analyzed using the SPSS Version 20.0 program (IBM SPSS Statistics for Windows, Version 20.0. Armonk, NY: IBM Corp).

## 3. Results

### 3.1. The General Characteristics of Participants

Among the 189 participants, 69 were premenopausal, 49 were perimenopausal, and 71 were postmenopausal. The mean age of participants was 48.97 years. The mean duration of HIV for the samples was 9.43 years and the mean duration of ART therapy was 8.31 years. About 5% of them had a CD4 cell count of less than 200 cells/mm^3^ and the majority (75.7%) fell under the III/IV stage of WHO HIV/AIDS clinical division (Table 1).

### 3.2. Mean Attitude towards Menopause by Menopausal Status

In the Kruskal–Wallis analysis, 14 items out of 35 were found with significant differences in three menopausal stages, and particularly higher score in the postmenopausal stage in a majority of the items (Table 2).

### 3.3. Total Attitude Scores by Participant Characteristics

The overall average attitude score toward menopause was 86.81 ± 10.79. The total attitude score was significantly higher for postmenopausal women (*p* = 0.007), older women (*p* = 0.001), women with education above primary (*p* = 0.050), and women who do not drink alcohol (*p* = 0.008). No statistically significant associations were observed with other variables investigated in the study (Table 3).

### 3.4. Factors Altering the Attitude towards Menopause

Multiple regression analysis showed that the older women (β = 0.173, *p* = 0.045), women with education above primary (β = 0.150, *p* = 0.034), and women who do not drink alcohol (β = 0.166, *p* = 0.018) were more likely to have more positive attitudes than their counter groups. However, menopausal status did not show any statistical association of menopausal status with the attitude towards menopause; when premenopause was used as a reference, *p*-values of peri- and post- menopuase were *p* > 0.05, *p* = 0.792, and *p* = 0.235, respectively (Table 4).

## 4. Discussion

Besides the fact that attitudes towards menopause plays significant role on its experience and severity, there is paucity of studies in this issue in Cambodia. This study aimed to investigate the attitude towards menopause along with the associated factors among WLWH in Cambodia.

First, the result of the study showed that participants held slightly negative attitudes toward menopause. Although ATM does not have a cut-point for positive or negative attitude, considering the score range from 35 to 140, the total attitude score measured by the ATM scale was 86.81 ± 10.79 for our participants. Participants in a study in Shiraz, Iran, had a positive attitude score of 102.7 ± 11.8 (of a possible 71–135), while Korean women had negative attitudes with a mean of 60.00 ± 10.12 (of a possible 33–108) when measured by the same scale; populations were seronegative in both studies [25,26]. Women with more negative attitudes toward menopause are found to experience more severe menopausal symptoms [14,15,24]. Because of the quantitative nature of the study, it was not possible to explore the intangible and abstract reasons behind the negatively perceived menopause. For example, variation in cultural beliefs is considered to cause variation in menopausal symptom experience and in attitude toward menopause [27]. Thus, further detailed qualitative study is recommended to investigate the culture-specific factors or social norms supporting the negative attitudes of Cambodian women.

Comparatively, postmenopausal women in our study demonstrated a higher attitude score and positive attitude toward menopause than women in other menopausal statuses (Table 2 and Table 3). This finding is consistent with other studies’ results measured with the same tool among seronegative participants [28,29]. Menopause should be approached with a biocultural approach. Native American Indians, for example, perceive menopause as a positive experience, and post-menopausal women are perceived as women with wisdom and are more respected within their communities [30]; in a case of the Turkish women, menopause is recognized as a freedom from feminine hygiene products or contraception/pregnancy [31]. It is unclear what Cambodian cultural factor makes postmenopausal women more positive for menopause, and further research is needed.

Furthermore, postmenopausal women supported positive statements like “Women are generally calmer and happier after the change of life than before” (*p* = 0.003), “Life is more interesting for a woman after menopause” (*p* = 0.001), “After the change of life, a woman gets more interested in community affairs than before” (*p* = 0.009), “A woman has a broader outlook on life after the change of life” (*p* = 0.031), “A woman gets more confidence in herself after the change of life” (*p* = 0.022), and “Many women think menopause is the best thing that ever happened to them” (*p* = 0.035). In contrast, premenopausal women significantly believed that “After the change of life, women often don’t consider themselves ‘real women’ any more” (*p* = 0.001). It can be interpreted that once women, irrespective of sero-status, undergo menopause, they find it less troubling than they imagined earlier. A woman who has not experienced this phenomenon is unsure about what to expect during menopause while those who had gone through this become less susceptible to menopause-related negative images and false stereotypes (if any) [29]. Similarly, as discussed in the study of Lydia et al. 2018 [32], compensation for the decline in cognitive–affective processes increases as an individual ages, which could result in the optimization of the life experiences and emotions. This gap in perceiving menopause between pre- and postmenopausal women can be reduced by consulting, encouraging, and guiding premenopausal women to acquire greater understanding on what menopause entails and to have a broader outlook on menopause by the health care providers. Health counselors can replace the fear of the unknown by accurate information, and can displace the negative stereotypes by healthy role models. Training packages with optimal information on menopause should be available to all aging women in order to prepare them for a healthy menopausal transition [24].

Though multiple factors are associated with age at menopause (AM), natural menopause is considered to occur between 50 and 52 years old [33,34,35]. For WLHIV, the average AM is reported to be lower than women generally, usually between 47 and 50 years old [17,36,37,38]. Consistent with those studies, it is seen that the mean AM for our participants is markedly earlier (48.47 ± 4.24 years) than the general menopause literature although the exact data is not available for the mean AM in Cambodia [39]. Lower AM means to remain in hypoestrogenism for a longer period of time, which could be associated with subsequent risk of dyslipidemia, insulin resistance, and osteopenia for those living with HIV due to the HIV infection itself and/or ART [40,41]. Larger studies identifying the mean AM among WLHIV in Cambodia are recommended. Additionally, the biochemical confirmation of menopause is warranted so that the appropriate counseling, screening, and management of menopause-related comorbidities can be applied [42].

Although more than half of our participants (66.1%) had primary or below primary education, higher education was significantly related to better attitudes in this study. The same finding was observed in other researches where well-educated women were seen holding more positive attitudes [13,43]. With the increasing level of education, women accept menopause as a natural developmental occurrence in life, like childhood and puberty [44]. Education increases women’s self-consciousness and informs them about the bodily changes and menopausal symptoms [45], thereby improving their attitude toward menopause. Furthermore, like prior studies among seronegative women, older age has been reported as a factor in a positive attitude toward menopause in the current study, too. Along with the increase in age, women are likely to go through the transition of life, making them less worried about menopause [13,29,32]. Hence, educating women about menopause in their early life before they enter the menopausal transition would be effective preparation for menopause along with positive attitudes toward it. The reason that drinking women have more negative attitudes is still unclear, thus it needs further exploration.

None of the HIV-related demographics were related to the attitude toward menopause in the current study. It suggests that the perception of Cambodian women regarding menopause is not affected by the HIV status. However, more detailed longitudinal investigation is necessary to validate this result. Assurances provided to women that menopausal symptoms are not features of a disease could help to alleviate their fears and improve attitudes toward menopause [46]. Health care providers in primary health centers, the most appropriate personnel to manage menopause because of their ability to combine menopause care with broader preventive health care measures for aging women, must be well trained and guided on delivering high-quality menopause care to the WLHIV [47]. Identifying what to expect in menopause is a need of Cambodian WLHIV, for which a comprehensive model of care should be developed that facilitates a tailored approach across the continuum of care and to improve their self-image. For this, better data are required to inform maximum provision of care and to upgrade their health attitudes in their post-reproductive years.

This study added the knowledge in the field that age and education level of WLHIV affect attitudes toward menopause as shown among sero-negative women; however, more studies in the various cultural groups are needed to validate the relationship of HIV infection and attitudes toward HIV menopause.

The cross-sectional nature of the study and the sample being confined to one specific hospital are limitations to the interpretation of this study result. However, since the hospital is a public hospital in the capital city of the country, there was a high diversity in the sociodemographic characteristics of the population without any potential bias in the recruitment process. Future study can include a larger number of women infected with HIV and use random sampling to have high levels of significance and representativeness.

## 5. Conclusions

This is the preliminary study on the attitude toward menopause of Cambodian women. Participants held slightly negative attitudes toward menopause. Postmenopausal women, older age, higher education, and a non-drinking habit were independently associated with a positive attitude toward menopause. Promoting positive attitudes toward menopause and preparing to experience menopause could help this population go through menopause with less suffering. Future research should study other possible predictors of menopause attitudes to assist this population in changing these attitudes for better understanding and easy transition to menopause. Future investigations, especially with qualitative studies, should give priority to better understanding of their quality of life, and the impact and the best behavioral strategies for management of menopause in this population.

## Figures and Tables

**Table 1:** Demographic characteristics by menopausal status (*n*= 189).

Characteristics		All (%) (*n* = 189)	Menopausal Status (*n*, %)		
			Premenopausal (*n* = 69) (36.5%)	Perimenopausal (*n* = 49) (25.9%)	Postmenopausal (*n* = 71) (37.6%)
Age Mean ± SD	40–49	121 (64.0)	65 (53.7)	35 (28.9)	21 (17.4)
	50–60	68 (36.0)	4 (5.9)	14 (20.6)	50 (73.5)
	(48.97 ± 5.77)		(44.72 ± 3.59)	(48.47 ± 4.24)	(53.45 ± 5.13)
Residence	Phnom Penh	111 (58.7)	40 (36.0)	29 (26.1)	42 (37.8)
	Province	78 (41.3)	29 (37.2)	20 (25.6)	29 (37.2)
Education	Primary and below	125 (66.1)	38 (30.4)	38 (30.4)	49 (39.2)
	Above primary	64 (33.9)	31 (48.4)	11 (17.2)	22 (34.4)
Employment status	Employed	124 (65.6)	51 (41.1)	31 (25.0)	42 (33.9)
	Unemployed	65 (34.4)	18 (27.7)	18 (27.7)	29 (44.6)
Monthly personal income	≤100 USD	112 (59.3)	32 (28.6)	28 (25.0)	52 (46.4)
	>100 USD	77 (40.7)	37 (48.1)	21 (27.3)	19 (24.7)
Marital status	Unmarried	11 (5.8)	4 (36.4)	3 (27.3)	4 (36.4)
	Married	68 (36.0)	30 (44.1)	21 (30.9)	17 (25.0)
	Divorced/Widowed/Separately living	110 (58.2)	35 (31.8)	25 (22.7)	50 (45.5)
Physical exercise	Never	71 (37.6)	26 (36.6)	17 (23.9)	28 (39.4)
	1–4 days/week	54 (66.1)	23 (42.6)	18 (33.3)	13 (24.1)
	5–7 days/week	64 (33.9)	20 (31.2)	14 (21.9)	30 (46.9)
Smoking	Yes	2 (1.1)	0 (0.0)	1 (50.0)	1 (50.0)
	No	187 (98.9)	69 (36.9)	48 (25.7)	70 (37.4)
Drinking	Yes	25 (13.2)	13 (52.0)	5 (20.0)	7 (28.0)
	No	164 (86.8)	56 (34.1)	44 (26.8)	64 (39.0)
Parity	1–2	162 (85.7)	65 (40.1)	44 (27.2)	53 (32.7)
	≥3	27 (14.3)	4 (14.8)	5 (18.5)	18 (66.7)
Abortion(s)	Yes	128 (67.7)	47 (36.7)	31 (24.2)	50 (39.1)
	No	61 (32.3)	22 (36.1)	18 (29.5)	21 (34.4)
Sexually active	Yes	72 (38.1)	33 (45.8)	23 (31.9)	16 (22.2)
	No	117 (61.9)	36 (30.8)	26 (22.2)	55 (47.0)
Perceived health status	0–10	6.98 ± 1.88	7.22 ± 1.88	7.20 ± 1.96	6.61 ± 1.80
Perceived life status	0–10	6.40 ± 2.40	6.49 ± 2.07	6.28 ± 2.62	6.40 ± 2.57
Duration of HIV	Mean ± SD	9.43 ± 4.04	9.49 ± 3.98	10.07 ± 4.10	8.93 ± 4.04
Duration of ART therapy	Mean ± SD	8.31 ± 3.45	8.20 ± 3.23	8.81 ± 3.41	8.08 ± 3.69
CD4 cell count	<200 cells/mm^3^	9 (4.8)	2 (22.2)	3 (33.3)	4 (44.4)
	200–499 cells/mm^3^	94 (49.7)	32 (34.0)	25 (26.6)	37 (39.4)
	≥500 cells/mm^3^	86 (45.5)	35 (40.7)	21 (24.4)	30 (34.9)
WHO HIV/AIDS clinical stage	I/II	46 (24.3)	18 (39.1)	12 (26.1)	16 (34.8)
	III/IV	143 (75.7)	51 (35.7)	37 (25.9)	55 (38.5)

**Table 2:** Mean attitude towards menopause by menopausal status (Kruskal–Wallis).

No.	Statements	All (*n* = 189)	Premeno Pausal a (*n* = 69)	Perimeno Pausal b (*n* = 49)	Postmeno Pausal c (*n* = 71)	χ^2^	*p*	Post-Hoc Test
1.	Women often use the change of life (*menopause*) as an excuse for getting attention	2.83 ± 1.15	2.69 ± 1.08	2.77 ± 1.17	3.00 ± 1.18	3.487	0.175	
2.	Unmarried women have a harder time than married women do at the time of the menopause	2.40 ± 1.13	2.55 ± 1.04	2.14 ± 1.14	2.44 ± 1.19	4.415	0.110	
3.	If the truth were really known, most women would like to have themselves a fling at this time in their lives.	1.25 ± 0.57	1.35 ± 0.61	1.22 ± 0.47	1.17 ± 0.59	7.430	0.024	a > c
4.	Women who have trouble with the menopause are usually those who have nothing to do with their time.	2.15 ± 1.07	2.26 ± 0.99	2.14 ± 1.10	2.04 ± 1.11	2.488	0.289	
5.	A woman should see a doctor during the menopause.	1.39 ± 0.72	1.49 ± 0.80	1.45 ± 0.87	1.25 ± 0.47	2.645	0.267	
6.	Woman in menopause is apt (likely) to do crazy things she herself does not understand.	2.82 ± 1.12	2.55 ± 1.02	2.65 ± 1.13	3.20 ± 1.13	13.819	0.001	c > a, b
7.	Women who have trouble in the menopause are those who are expecting it.	1.29 ± 0.59	1.36 ± 0.66	1.20 ± 0.41	1.28 ± 0.61	1.644	0.443	
8.	The thing that causes women all their trouble at menopause is something they can’t control— changes inside their bodies.	2.04 ± 1.00	2.14 ± 0.93	1.94 ± 1.05	2.01 ± 1.04	2.475	0.291	
9.	A good thing about the menopause is that a woman can quit worrying about getting pregnant.	3.79 ± 0.63	3.67 ± 0.82	3.82 ± 0.57	3.90 ± 0.42	4.572	0.102	
10.	Menopause is a mysterious thing which most women don’t understand.	2.59 ± 1.21	2.58 ± 1.14	2.39 ± 1.22	2.75 ± 1.26	2.596	0.275	
11.	Woman is concerned about how her husband will feel towards her after the menopause.	2.25 ± 1.07	2.17 ± 1.00	2.31 ± 1.06	2.30 ± 1.14	0.427	0.809	
12.	Going through the menopause really does not change a woman in any important way.	2.52 ± 1.07	2.36 ± 0.97	2.59 ± 1.02	2.63 ± 1.20	2.663	0.265	
13.	Menopause is one of the biggest changes that happens in a woman’s life.	1.86 ± 0.98	1.96 ± 0.95	1.82 ± 0.99	1.80 ± 1.01	1.800	0.408	
14.	A woman’s body may change in menopause, but otherwise she doesn’t change much.	3.29 ± 0.73	3.32 ± 0.53	3.20 ± 0.91	3.32 ± 0.77	0.551	0.761	
15.	The only difference between a woman who has not been through the menopause and one who has, is that one menstruates and the other doesn’t.	3.08 ± 1.15	3.10 ± 1.07	3.00 ± 1.17	3.11 ± 1.21	0.540	0.765	
16.	Women are generally calmer and happier after the change of life (*after menopause*) than before.	2.84 ± 1.01	2.54 ± 0.95	2.98 ± 0.92	3.03 ± 1.06	11.516	0.003	b, c > a
17.	After the change of life (*after menopause*), a woman feels freer to do things for herself.	3.30 ± 0.86	3.17 ± 0.87	3.33 ± 0.85	3.41 ± 0.85	3.819	0.148	
18.	Women worry about losing their minds during the menopause.	2.19 ± 0.94	2.13 ± 0.84	1.98 ± 0.99	2.39 ± 0.96	6.279	0.043	c > a, b
19.	After the menopause, a woman is more interested in sex than she was before.	1.33 ± 0.69	1.46 ± 0.81	1.37 ± 0.64	1.17 ± 0.56	9.000	0.010	a, b > c
20.	It’s no wonder women feel “down in the dumps” at the time of the menopause.	2.42 ± 1.05	2.29 ± 0.96	2.31 ± 0.98	2.63 ± 1.15	4.171	0.124	
21.	After the change of life, a woman gets more interested in community affairs than before.	3.21 ± 0.81	3.01 ± 0.80	3.29 ± 0.71	3.35 ± 0.86	9.358	0.009	b, c > a
22.	Women think of menopause as the beginning of the end.	2.62 ± 1.24	2.78 ± 1.15	2.29 ± 1.17	2.70 ± 1.34	5.178	0.075	
23.	Life is more interesting for a woman after the menopause.	2.83 ± 0.92	2.51 ± 0.90	3.06 ± 0.85	2.99 ± 0.92	14.661	0.001	b, c > a
24.	Women generally feel better after the menopause than they were for years.	2.77 ± 1.00	2.59 ± 0.91	2.80 ± 1.02	2.92 ± 1.05	4.710	0.095	
25.	After the change of life (*after menopause*), women often don’t consider themselves “real women” any more.	2.84 ± 1.19	3.06 ± 1.07	2.29 ± 1.15	3.00 ± 1.22	14.519	0.001	a, c > b
26.	A woman has a broader outlook on life after the change of life (*after menopause*).	2.89 ± 0.95	2.70 ± 0.83	3.08 ± 0.95	2.94 ± 1.04	6.888	0.031	b, c > a
27.	A woman gets more confidence in herself after the change of life (*after menopause*).	3.01 ± 0.93	2.81 ± 0.84	3.20 ± 0.89	3.06 ± 1.00	7.617	0.022	b, c > a
28.	Menopause is an unpleasant experience for a woman.	2.15 ± 1.03	2.13 ± 1.03	2.14 ± 0.98	2.17 ± 1.07	0.037	0.982	
29.	Women often get self-centered at the menopause.	3.24 ± 0.75	2.93 ± 0.75	3.37 ± 0.73	3.45 ± 0.67	19.868	<0.001	b, c > a
30.	Menopause is a disturbing thing which most women naturally dread.	2.36 ± 1.12	2.23 ± 0.96	2.04 ± 1.04	2.70 ± 1.24	10.063	0.006	c > a, b
31.	After the change of life (*after menopause*), a woman has a better relationship with her husband.	2.25 ± 0.95	2.29 ± 0.86	2.43 ± 1.04	2.07 ± 0.96	3.825	0.148	
32.	It’s not surprising that most women get disagreeable during the menopause.	2.31 ± 1.06	2.26 ± 0.98	2.16 ± 1.07	2.46 ± 1.12	2.540	0.282	
33.	In truth, just about every woman is depressed about the change of life (*after menopause*).	2.63 ± 1.13	2.67 ± 1.02	2.29 ± 1.19	2.85 ± 1.14	7.166	0.027	a, c > b
34.	Women should expect some trouble during the menopause.	1.74 ± 0.79	1.64 ± 0.71	1.67 ± 0.69	1.87 ± 0.91	2.121	0.348	
35.	Many women think menopause is the best thing that ever happened to them.	2.32 ± 1.08	2.13 ± 1.03	2.22 ± 1.12	2.58 ± 1.05	6.654	0.035	c > a, b

**Table 3:** Total attitude scores by participant characteristics.

Characteristics		Frequency (*n*)	Total Attitude Score (86.81 ± 10.79)	F or T (Duncan)	*p*
Menopausal status	Premenopausal	69	84.90 ± 9.95	5.048	0.007
	Perimenopausal	49	84.94 ± 10.15		
	Postmenopausal	71	89.96 ± 11.40		
Age	40–49	121	84.81 ± 9.48	3.461	0.001
	50–60	68	90.37 ± 12.08		
Residence	Phnom Penh	111	86.78 ± 10.55	1.573	0.969
	Province	78	86.85 ± 11.19		
Education	Primary and below	125	85.71 ± 11.03	1.174	0.050
	Above primary	64	88.95 ± 10.05		
Employment status	Employed	124	86.45 ± 10.35	0.259	0.530
	Unemployed	65	87.49 ± 11.63		
Monthly personal income	≤100 USD	112	86.86 ± 11.10	0.356	0.942
	>100 USD	77	86.74 ± 10.40		
Marital status	Unmarried	11	82.73 ± 8.39	1.167	0.313
	Married	68	87.90 ± 10.27		
	Divorced/Widowed/Separately living	110	86.55 ± 11.27		
BMI	Underweight	27	89.22 ± 10.73	1.311	0.272
	Normal	101	86.27 ± 10.52		
	Overweight	31	84.58 ± 10.78		
	Obesity	30	88.81 ± 11.57		
Physical exercise	Never	71	85.66 ± 9.84	0.821	0.442
	1–4 days/week	54	86.85 ± 11.53		
	6–7 days/week	64	88.81 ± 11.18		
Smoking	Yes	2	86.50 ± 13.44	0.037	0.968
	No	187	86.81 ± 10.80		
Drinking	Yes	25	81.48 ± 11.23	0.183	0.008
	No	164	87.62 ± 10.52		
Parity	1–2	162	86.23 ± 10.83	0.166	0.073
	≥3	27	90.26 ± 10.07		
Abortion(s)	Yes	128	86.30 ± 10.94	0.004	0.353
	No	61	87.87 ± 10.49		
Sexually active	Yes	72	86.29 ± 10.38	0.180	0.606
	No	117	87.13 ± 11.07		
CD4 cell count	<200 cells/mm^3^	9	86.55 ± 10.41	0.098	0.907
	200–499 cells/mm^3^	94	87.16 ± 10.62		
	≥500 cells/mm^3^	86	86.45 ± 11.12		
WHO HIV/AIDS clinical stage	I/II	46	84.57 ± 12.11	1.864	0.105
	III/IV	143	87.53 ± 10.27		

**Table 4:** Multiple regression analysis for factors altering the attitude towards menopause.

Variables	β	SE	t	*p*
Menopausal status ^a^				
Perimenopause	−0.022	2.004	−0.264	0.792
Postmenopause	0.115	2.146	1.192	0.235
Age (50–60) ^b^	0.173	1.917	2.020	0.045
Education (Above primary)	0.150	1.604	2.134	0.034
Drinking (No)	0.166	2.218	2.384	0.018
Constant			56.113	<0.001
Adj R^2^	0.101			
F	5.217			
*p*	<0.001			

*Note.* Confounding variables (only the significant ones from Table 3) age, education and drinking were controlled. ^a^ Premenopause as reference; ^b^ age 40–49 as reference.

## Data Availability

The data is available from the corresponding author, upon reasonable request.
